# Fibrinogen production is enhanced in an in-vitro model of non-alcoholic fatty liver disease: an isolated risk factor for cardiovascular events?

**DOI:** 10.1186/s12944-015-0069-3

**Published:** 2015-08-10

**Authors:** Emily N. W. Yeung, Philipp Treskes, Sarah F. Martin, Jonathan R. Manning, Donald R. Dunbar, Sophie M. Rogers, Thierry Le Bihan, K. Ann Lockman, Steven D. Morley, Peter C. Hayes, Leonard J. Nelson, John N. Plevris

**Affiliations:** Hepatology Laboratory, Division of Health Sciences, University of Edinburgh, Chancellor’s Building, 49 Little France Crescent, Edinburgh, EH16 4SB UK; Kinetic Parameter Facility, SynthSys, Centre for Synthetic and Systems Biology, University of Edinburgh, C.H. Waddington Building, The Kings Buildings, Edinburgh, EH9 3JD UK; Bioinformatics Team, University/BHF Centre for Cardiovascular Science, University of Edinburgh, Queen’s Medical Research Institute, 47 Little France Crescent, Edinburgh, EH16 4TJ UK

**Keywords:** Systems biology, Proteomics, Microarray, STRING, Non-alcoholic fatty liver disease, Cardiovascular disease, Metabolic syndrome, C3A cells

## Abstract

**Background:**

Cardiovascular disease (CVD) remains the major cause of excess mortality in patients with non-alcoholic fatty liver disease (NAFLD). The aim of this study was to investigate the individual contribution of NAFLD to CVD risk factors in the absence of pathogenic influences from other comorbidities often found in NAFLD patients, by using an established *in-vitro* model of hepatic steatosis.

**Methods:**

Histopathological events in non-alcoholic fatty liver disease were recapitulated by focused metabolic nutrient overload of hepatoblastoma C3A cells, using oleate-treated-cells and untreated controls for comparison. Microarray and proteomic data from cell culture experiments were integrated into a custom-built systems biology database and proteogenomics analysis performed. Candidate genes with significant dysregulation and concomitant changes in protein abundance were identified and STRING association and enrichment analysis performed to identify putative pathogenic pathways.

**Results:**

The search strategy yielded 3 candidate genes that were specifically and significantly up-regulated in nutrient-overloaded cells compared to untreated controls: fibrinogen alpha chain (2.2 fold), fibrinogen beta chain (2.3 fold) and fibrinogen gamma chain (2.1 fold) (all rank products pfp <0.05). Fibrinogen alpha and gamma chain also demonstrated significant concomitant increases in protein abundance (3.8-fold and 2.0-fold, respectively, *p* <0.05).

**Conclusions:**

*In-vitro* modelling of NAFLD and reactive oxygen species formation in nutrient overloaded C3A cells, in the absence of pathogenic influences from other comorbidities, suggests that NAFLD is an isolated determinant of CVD. Nutrient overload-induced up-regulation of all three fibrinogen component subunits of the coagulation cascade provides a possible mechanism to explain the excess CVD mortality observed in NAFLD patients.

**Electronic supplementary material:**

The online version of this article (doi:10.1186/s12944-015-0069-3) contains supplementary material, which is available to authorized users.

## Background

Non-Alcoholic Fatty Liver Disease (NAFLD) has been traditionally regarded as the consequence of a high-fat western diet and sedentary lifestyle [1, 2]. Its increasing worldwide prevalence, however, suggests that NAFLD is more than a lifestyle disease. Studies have identified susceptibility genes and genetic polymorphisms that associate with development and severity of NAFLD [3]. This may explain the differences in demographic data observed in Asia compared to elsewhere in the world, including a younger age distribution and a higher proportion of patients who are judged lean by Body Mass Index, but show an altered metabolic profile associated with obesity [4, 5].

Cardiovascular disease (CVD) remains one of the major causes of excess mortality in NAFLD patients [6–8]. The term “Metabolic Syndrome” (MetS) describes a clinical cluster of diseases that tend to aggregate in individuals over time, including central obesity, hypertension, impaired fasting glucose and dyslipidemia [9–11]. Together these components create a pro-atherogenic environment that is postulated to accelerate atherosclerosis and increase the risk of cardiovascular diseases. Whilst the definition of MetS does not presently include NAFLD, the association of atherosclerotic markers, such as carotid artery wall thickness, with NAFLD has been reported previously [12, 13]. Further attempts to understand the possible causative role of NAFLD in CVD have used statistical models to exclude atherogenic contributions from traditional CVD risk factors and other components of MetS [14, 15]. However, to date, there are no experimental genomic or proteomic studies that examine specifically whether NAFLD is an isolated risk factor for CVD in absence of other components of MetS, or if these conditions have a common cause.

Previously, we developed an *in vitro* model of cellular steatosis by exposing hepatoblastoma C3A cells to nutrient overload (treatment with lactate, pyruvate, octanoate and ammonia), which reproduces the characteristic pathophysiological changes found in NAFLD, including intracellular triglyceride accumulation and reactive oxygen species (ROS) formation [16]. This model allows the opportunity to assess the individual contribution of NAFLD to CVD risk factors in the absence of pathogenic influences from other comorbidities often found in NAFLD patients.

In the present study, changes in hepatoblastoma C3A gene transcription and protein expression in response to cellular steatosis and ROS formation induced specifically by nutrient overload were assembled into a custom-built data portal allowing evaluation of integrated transcriptomic and proteomic data to identify gene products potentially involved in pathogenic pathways. Candidates showing consistently greater than two fold alterations in specific nutrient overload-mediated gene transcription and protein expression were subjected to further analysis by the Search Tool for the Retrieval of Interacting Genes/Proteins (STRING) v9.1 database (http://string-db.org) and enrichment analysis to identify predicted functional partners and pathogenic pathways contributing potentially to a pro-atherogenic environment.

## Methods

### Cell culture, treatment and sample collection

Hepatoblastoma C3A cells (ATCC® CRL-10741TM, LGC Standards, Teddington, UK) were cultured as previously described [16]. Briefly, cell cultures were treated either with the combination of lactate, pyruvate, octanoate and ammonia (LPON), oleate (OLE), or untreated controls. Both octanoate and oleate readily diffuse into mitochondria to promote efficient ß-oxidation and lipid accumulation, but while OLE treatment causes simple cellular steatosis, LPON treatment induces both ROS formation and mitochondrial dysfunction, in addition to steatosis, typically seen in NAFLD [16]. C3A cells were treated in three separate experiments either with LPON, OLE, or untreated controls and processed for transcriptomic or proteomic analysis as described in the following sections.

### Sample preparation and transcriptomics

Cells were washed twice in cold PBS and transferred to cold RNALater® (Life Technologies, Paisley, UK) for overnight incubation at 4 °C. Afterwards, RNA was isolated with an RNAqueous®-4PCR kit (Life Technologies) and subsequently amplified and biotinylated with an Illumina® TotalPrep RNA Amplification kit (Life Technologies), following the manufacturer’s instructions. RNA expression was measured by hybridization to the Illumina® Whole Human Genome BeadChip H12 Microarray (Illumina United Kingdom, Essex, UK). Data were extracted through the GCOS software (Affymetrix UK Ltd., High Wycombe, UK). CELfiles were used for additional data processing and imported into Bioconductor [17] to examine differences between LPON- and OLE-treated groups and untreated controls. Data were normalized by robust multi-array average (RMA) in the Oligo module (http://www.bioconductor.org/packages/2.0/bioc/html/oligo.html). Gene ontology and Kyoto Encyclopedia of Genes and Genomes (KEGG) pathway enrichment analysis was performed with the DAVID tool [18, 19] on genes that were significantly differentially expressed. Data was statistically analyzed with the bioconductor Limma package [20].

### Sample preparation and proteomics

Protein extraction was performed as previously described [21]. Briefly, samples were denatured in 8 M urea, reduced by incubating with dithiothreithol prior to cysteine alkylation with iodoacetamide and overnight digestion with 8 μg trypsin. Protein concentrations were estimated by Bradford protein assay (Thermo Scientific, Rockford, IL, USA) on a 10 μl sample, diluted to 2 M Urea and quantified against a BSA standard curve. 4 μg peptide samples were acidified (1 % formic acid), centrifuged and cleaned using Stagetips [22], dried by SpeedVac, and stored at −20 °C.

2 μg peptide samples were analysed in a randomised sequence by capillary-HPLC- MSMS as described previously [23], using an on-line system consisting of a micro-pump (1200 binary HPLC system, Agilent, UK) coupled to a hybrid LTQ-Orbitrap XL instrument (Thermo-Fisher, Leicestershire, UK). Acetonitrile and water were HPLC quality (Fisher). Formic acid was Suprapure 98-100 % (Merck, Darmstadt, Germany) and trifluoroacetic acid was 99 % purity sequencing grade. LC-MSMS label-free quantification was performed using Progenesis 4.0 (Nonlinear Dynamics, Newcastle upon Tyne, UK) as described previously [24]. Multicharged (2+,3+,4+) ion intensities were extracted from LC-MS files and MSMSdata were searched using Mascot Version 2.4 (Matrix Science Ltd, London, UK) against the Homo Sapiens subset of the NCBI protein database (12/01/2011; 34,281 sequences) using a maximum missed-cut value of 2, variable oxidation (M), N-terminal protein acetylation and fixed carbamidomethylation (C); precursor mass tolerance was 7 ppm and MSMS tolerance 0.4 Da. The significance threshold (p) was < 0.05 (MudPIT scoring). A minimum peptide cut off score of 20 was set, corresponding to <3 % global false discovery rate (FDR) using a decoy database search. Proteins identified and quantified with two or more peptide sequences were retained. A two-tailed t-test for independent samples or biological triplicates was performed on arcsinh transformed, normally distributed intensity data.

### Data mining and candidate gene identification

**Fig. 1:**
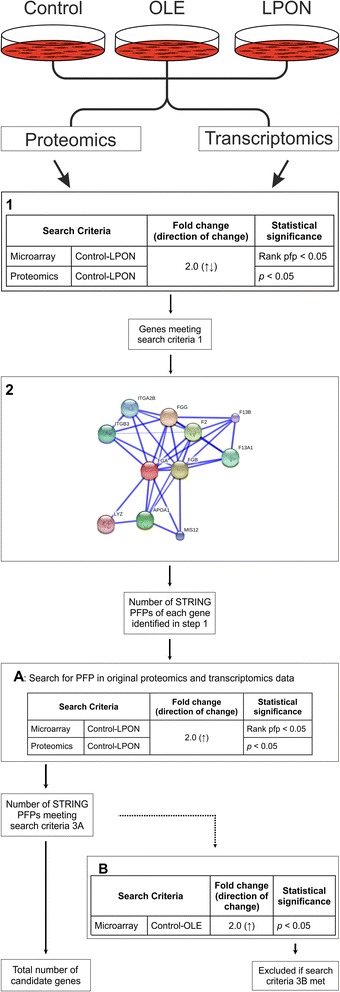
Strategy for data mining and candidate gene identification. Step 1: C3A cell cultures treated with LPON, OLE or untreated controls, as described under ‘Methods’, were subject to transcriptomic or proteomic analysis and data screened for candidate genes specifically up-regulated by nutrient overload using the screening criteria outlined in Box 1. Step 2: Predicted functional partners of primary candidates showing >2.0 fold nutrient overload-induced increases in gene transcripts and protein abundance were identified by STRING network associations. Step 3: Primary gene candidates, identified in step 1, and their predicted functional partners, identified in step 2, were grouped by enrichment analysis according to their functional annotations to identify representation of common biological processes among the candidate genes. Further details of these bioinformatics procedures are described under ‘Methods’.

Changes in hepatoblastoma C3A gene transcription and protein expression in response to cellular steatosis induced by nutrient overload, compared with OLE-treatment and untreated controls, were analysed in a custom built bioinformatics data mining tool established by the BHF Centre of Research Excellence Bioinformatics Team at the Queen’s Medical Research Institute at the University of Edinburgh. This allows evaluation of integrated transcriptomic and proteomic data, by matching differential gene transcription with altered protein abundance, to identify gene products potentially involved in pathogenic pathways. Genes of interest were analysed in a stepwise approach outlined in Fig. 1 to identify genes showing a consistently greater than 2.0 fold increase in both gene transcription and protein abundance. To focus on the specific effects of steatosis with ROS formation and mitochondrial dysfunction and to eliminate candidates up-regulated by simple steatosis, genes showing similar > 2.0 fold increases in expression in both OLE- and LPON-treated cells compared to untreated controls were excluded from the analysis.

### STRING Network Associations

Predicted functional partners of candidate genes induced specifically in response to nutrient overload were identified using the Search Tool for the Retrieval of Interacting Genes/Proteins (STRING) v9.1 database (http://string-db.org). Only interactions in Homo sapiens with a probabilistic confidence score ≥0.900, corresponding to a "highest-confidence" network, were considered in this study. STRING PFPs were cross-validated against the original transcriptomic and proteomic data for differential expression and protein abundance, using similar criteria (>2.0 fold increase in LPON-treated cells, excluding PFPs with >2.0 fold increase in OLE-treated cells: see Fig. 1) in order to identify candidate genes that had otherwise been excluded by the more stringent primary search strategy.

### Enrichment analysis

To identify dysregulated pathways and biological processes contributing potentially to a pathogenic phenotype, candidate genes differentially expressed in response to nutrient overload and their PFPs were grouped according to their functional annotations with data from the Gene Ontology (GO) and Kyoto Encyclopedia of Genes and Genomes (KEGG) database via the Gene Ontology enRIchment anaLysis and visuaLizAtion (GOrilla) tool (http://cbl-gorilla.cs.technion.ac.il) [25, 26]. Only dysregulations with p < 0.05 were considered in this study, with p-values being corrected for multiple testing using the Benjamini and Hochberg method [27]. Enrichment was based on gene ranking, which was indicated by the STRING analysis confidence score.

## Results

Bioinformatics, using the screening criteria defined in step 1 of Fig. 1, identified 2 genes that showed a >2-fold increase in both gene transcription and protein abundance specifically in response to nutrient overload, namely fibrinogen alpha chain and farnesyl-diphosphate farnesyltransferase (Tables 1 and 2). These were selected for STRING association and enrichment analysis to identify predicted functional partners and pathogenic pathways as described below. Several other genes demonstrated a >2-fold increase in specific nutrient overload- induced gene transcription, but lacked corroborative proteomics data and consequently were not used for primary STRING association and enrichment analysis. No examples were found of any gene showing a >2-fold reduction in gene transcription and protein abundance, supporting either unchanged or increased gene expression specifically in response to nutrient overload.

**Table 1:** Microarray data for candidate genes specifically up-regulated by nutrient overload

Entrez gene ID	Accession	Gene Symbol	Description	Control	LPON		Control-LPON		OLE		Control-OLE	
				Max Mean value	Max Mean value	Direction of change wrt control	Maximum FC	RP pfp	Max Mean value	Direction of change wrt control	Max Mean value	RP pfp
2222		FDFT	farnesyl-diphosphate farnesyltransferase 1	1055.01	2501.65	↑	2.37121	0	1558.93	↑	1.47764	0.0194
2243	NP_000499	FGA	Fibrinogen alpha chain	3686.45	7280.47	↑	2.24956	0.0002	6716.9	↑	1.97551	0.0009

FGA and FDFT were identified as candidate genes showing > 2.0 fold specific up-regulation of gene expression and protein abundance (see Table 2) in LPON-treated cells compared to untreated controls.

*LPON* Lactate, pyruvate, octanoate and ammonia, *FC* fold change, *RP pfp* Rank products estimated percentage of false positive predictions; *FGA* Fibrinogen alpha chain, *FDFT* Farnesyl-diphosphate farnesyltransferase 1.

**Table 2:** Proteomics data for candidate genes specifically up-regulated by nutrient overload

	Gene	Unique			Ratio
Accession	Symbol	Peptides	Description	p value	LPON/ctrl
NP_004453	FDFT	3	Farnesyl-diphosphate farnesyltransferase 1	0.015711376	3.22
NP_000499	FGA	1	Fibrinogen alpha chain	0.00913822	3.81

FGA and FDFT were identified as candidate genes showing > 2.0 fold specific up-regulation of gene expression (see Table 1) and protein abundance in LPON-treated cells compared to untreated controls.

For abbreviations: see Table 1.

### Fibrinogen alpha chain (FGA)

FGA gene expression was significantly higher in LPON-treated cells compared to untreated controls by 2.3 fold (rank products pfp = 0.0002). The FGA protein product was also more abundant in LPON-treated cells (3.8 fold change, p = 0.009).

STRING network analysis (step 2, Fig. 1) identified 32 STRING PFPs of FGA in *Homo sapiens* with a probabilistic confidence score ≥ 0.900 (Supplementary material; Additional File 1). Expression of four out of 32 PFPs were up-regulated by >2 fold in LPON-treated cells compared to untreated controls (rank products pfp <0.05). This included the remaining two fibrinogen beta (FGB) and fibrinogen gamma (FGG) chains, fibrinogen-like 1 (FGL1), and cystatin C (CST3) (Table 3). These 4 genes were not identified in the primary analytical strategy either because no proteomics data was available (FGB, FLG1, CST3; Table 4) or the differential protein abundance did not quite reach the primary fold change search criteria and/or statistical significance (FGG, fold change 1.97462, p = 0.07, Table 4).Of these up-regulated PFPs, FGL1 and CST3 were subsequently excluded from further analysis, as transcription of both genes was also up-regulated by more than 2-fold in OLE-treated cells compared to untreated controls (Table 3). In contrast, FGG and FGB, along with FGA identified in the primary screen, were up-regulated to a much lesser extent in OLE-treated compared to LPON-treated cells (all less than 2 fold-change, see Tables 1 and 4). In total, three genes of the coagulant cascade (FGA, FGG, and FGB) met the final inclusion criteria as being specifically up-regulated in response to nutrient overload.

**Table 3:** Microarray data for predicted functional partners of FGA up-regulated by nutrient overload

Entrez gene ID	Accession	Gene symbol	Description	Control	LPON		Control-LPON		OLE		Control-OLE	
				Maximum mean value	Maximum mean value	Direction of change wrt control	Maximum FC	RP pfp	Maximum mean value	Direction of change wrt control	Maximum FC	RP pfp
2266	NP_000500	FGG	Fibrinogen gamma chain	4612.12	1227.85	↑	2.10864	0.003	7458.56	↑	1.61716	0
2244	NP_001171670	FGB	Fibrinogen beta chain	644.648	1505.62	↑	2.33556	0.0018	1239.23	↑	1.92234	0.02
2267	NP_004458	FGL1	Fibrinogen-like 1	400.579	1086.52	↑	2.43005	0.003	884.484	↑	2.20802	0.0178
1471	NP_000090	CST3	Cystatin C	2153.68	5232.34	↑	2.42948	0	4730.95	↑	2.19668	0

>2 fold up-regulation in gene expression of FGG, FGB, FGL1 and CST3 were observed in LPON-treated cells compared to untreated controls.

For abbreviations: see Table 1.

**Table 4:** Proteomics data for predicted functional partners of FGA up-regulated by nutrient overload

	Gene	Unique			Ratio
Accession	Symbol	Peptides	Description	*P* value	LPON/ctrl
NP_000500	FGG	3	Fibrinogen gamma chain	0.072855965	1.97

For abbreviations: see Table 1.

Enrichment analysis of FGA and its STRING-predicted functional partners by gene ontology revealed platelet activation, coagulation, and hemostasis as significantly over-represented biological processes (Table 5 ).

**Table 5:** Enrichment analysis based on the number of predicted functional partners of FGA indicated by STRING analysis

GO term	Description	*P*-value	FDR q-value	Enrichment (N, B, n, b)
GO:0030168	Platelet activation	1.43E-05	1.96E-02	3.00 (33,11,8,8)
GO:0051592	Response to calcium ion	1.83E-04	1.26E-01	11.00 (33,3,3,3)
GO:0050817	Coagulation	3.02E-04	1.38E-01	1.55 (33,16,20,15)
GO:0007599	Chemostasis	3.02E-04	1.04E-01	1.55 (33,16,20,15)
GO:0007596	Blood coagulation	3.02E-04	8.30E-02	1.55 (33,16,20,15)
GO:0001775	Cell activation	4.88E-04	1.12E-01	2.36 (33,14,8,8)
GO:0006887	Exocytosis	5.88E-04	1.15E-01	2.89 (33,10,8,7)
GO:0002576	Platelet degranulation	5.88E-04	1.01E-01	2.89 (33,10,8,7)
GO:0032940	Secretion by cell	5.88E-04	8.96E-02	2.89 (33,10,8,7)

Enrichment was calculated as (b/n) / (B/N), where N is the total number of partners including FGA; B is the total number of genes associated with a specific GO term; n is the number of genes in the STRING partner list, as ranked by confidence score or in the target set when appropriate b is the number of genes in the intersection.

### Farnesyl-diphosphate farnesyltransferase 1 (FDFT1)

Gene expression of FDFT1, which catalyses the first committed step in sterol synthesis on the pathway to cholesterol, was up-regulated by 2.4 fold (rank products pfp = 0) in LPON-treated cells compared to untreated controls. A concomitant increase in protein abundance by 3.2 fold (p = 0.02) in LPON-treated cells was also recorded. 10 STRING PFPs of FDFT1 in *Homo sapiens* with a probabilistic confidence score ≥0.900 were identified (Supplementary material; Additional File 2); however none of these genes satisfied the criteria of >2.0 fold change in gene expression (Rank products pfp <0.05) when comparing LPON-treated cells to untreated controls.

## Discussion

CVD remains a major cause of excess mortality in NAFLD patients [6–8]. In the present study, we sought to clarify the possible causative role of NAFLD in CVD by identifying gene and protein dysregulation using an *in vivo* model of cellular steatosis. Use of an *in vitro* model is particularly relevant as it enables experimental strategies to be designed to exclude pathogenic influences from conditions, such as diabetes mellitus and hypertension, which commonly co-exist with NAFLD [28].

**Fig. 2:**
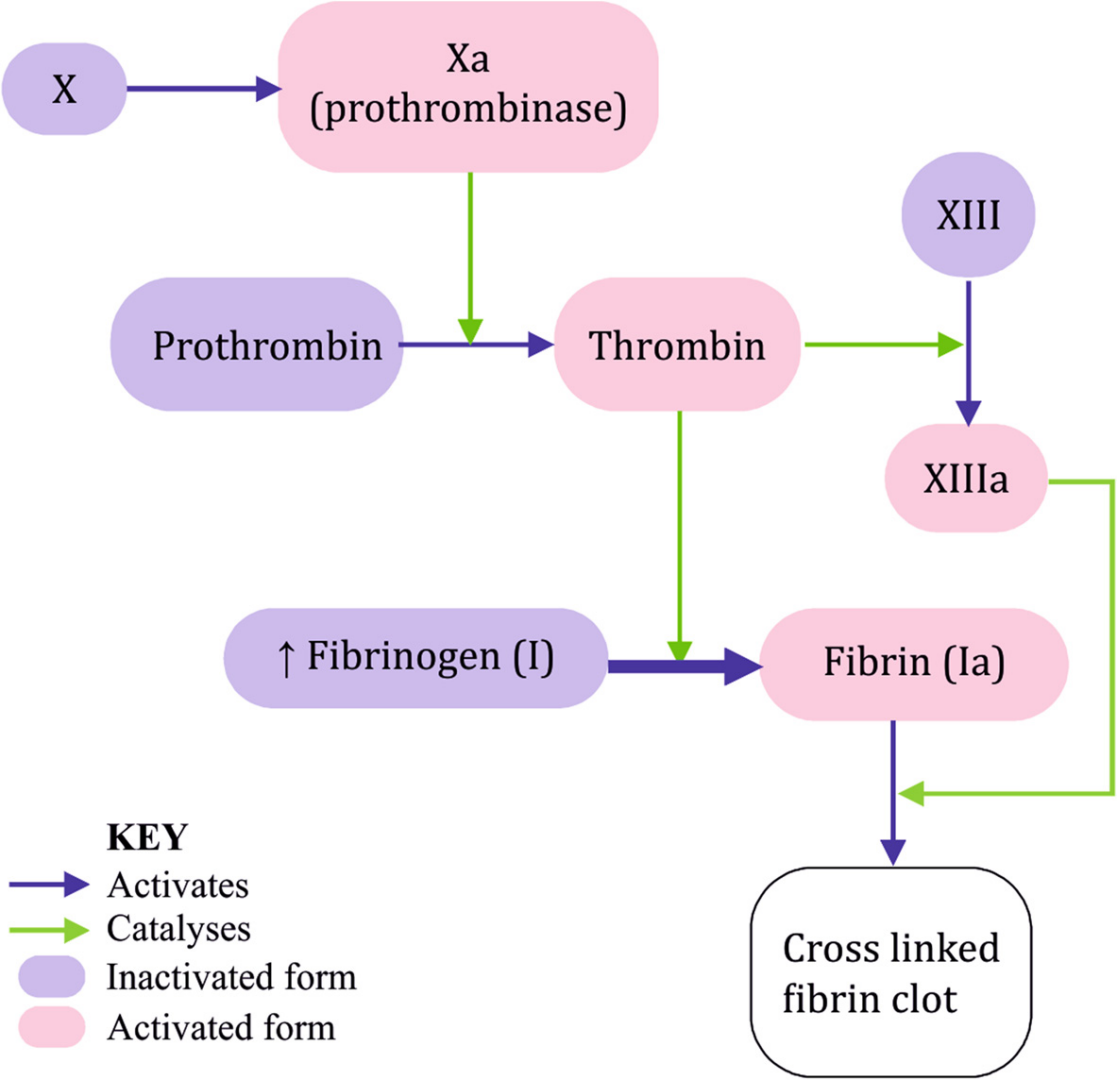
Common pathway of the coagulation cascade. Common pathway of the coagulation cascade, showing how fibrinogen (factor I) as a precursor of fibrin (Ia), contributes to the subsequent formation of a stable fibrin clot.

Fibrinogen is produced in the liver and is made up of alpha, beta, and gamma chains in a 1:1:1 ratio. We found similar up-regulation in gene expression of all three fibrinogen subunits (see Tables 1 and 3), which provides a potential mechanism to explain the excess CVD mortality observed in NAFLD patients. In the common pathway of the coagulation cascade, fibrinogen (factor I) is a precursor of fibrin (Ia), which contributes to the subsequent formation of a stable fibrin clot (see Fig. 2). An increased plasma fibrinogen level is therefore prothrombotic and its potential clinical application as a surrogate biomarker for predicting future CVD events is currently being explored [29, 30]. Two large-scale studies, which together included 400,880 patients, concluded that fibrinogen is a reliable predictor of cardiovascular mortality [31, 32]. Such a correlation persists even after adjustment for other established risk factors of CVD known to associate with fibrinogen levels, including total cholesterol and blood pressure. This suggests that there is an independent association between plasma fibrinogen levels and CVD. Therefore NAFLD, through up-regulation of fibrinogen coagulation factor, is an independent risk factor of CVD, potentially by enhancing clot strength [33]. This is logical, because liver is the site of synthesis of most coagulation factors, and therefore pathologies of the liver would most probably contribute to systemic cardiometabolic dysregulation. Indeed, elevated plasma fibrinogen is frequently reported as an additional and independent cardiovascular risk factor in a clinical context of MetS, with a significant association with the severity of hyperinsulinaemia [34, 35]. To our knowledge, our *in vitro* approach is unique in providing a controlled environment for isolating events that occur specifically in the hepatocytes, independent of the influence exerted by other disease variables forming the clinical cluster of MetS. This allows us to identify hepatocyte dysfunction as the pathophysiological culprit of increased cardiovascular risk observed in these individuals.

### Difference between OLE and LPON-treated cells

Up-regulation in the genetic expressions of all 3 fibrinogen chains was consistently more dramatic in LPON-treated compared OLE-treated cells, the latter of which models simple cellular steatosis. In contrast, LPON treatment additionally induced ROS formation and mitochondrial dysfunction [16] Thus, we hypothesize that dysregulation of gene expression in LPON-treated cells was made more dramatic by oxidative stress due to free radical formation.

Previous cross-sectional studies have demonstrated increased blood fibrinogen concentrations in response to mild pulmonary inflammation in healthy humans caused by diesel exhaust particulates, a major constituent of city air pollution [36, 37]. In a previous double-blinded randomised crossover study, diesel exhaust inhalation has also been shown to mediate excess CVD risk caused by air pollution by increasing thrombus formation following platelet activation secondary to ROS formation and oxidative stress [38]. This supports the notion that multiple mechanisms intersecting at the level of ROS formation may contribute to excess CVD risk. However, while ROS-formation in C3A cells after exposure to nanoparticles has been confirmed in our laboratory, such observations should be taken with caution as the mechanism of free radical generation may be different in particulate-induced and steatosis-induced conditions [39]. Thus, while ROS formation observed after inhalation of diesel exhaust arises from the alteration of redox potential by particulates and inflammatory cells [40], in our model of NAFLD, ROS are formed endogenously due to nutrient excess [16].

In conclusion, this study presents evidence supporting the hypothesis of NAFLD being an isolated determinant of CVD, as use of a proven *in vitro* model of NAFLD eliminates the confounding influences of other comorbidities found in NAFLD patients. This supports the notion that the liver is a source of pro-coagulant molecules which contributes to the risk of thrombosis and enhanced CVD risk in the NAFLD population. However, in using an *in vitro* model examines only one aspect of the concomitant pathophysiological mechanisms operating *in vivo*. Other factors operating in concert may play significant roles in modifying the systemic response to the observed cellular effects, for example the role of visceral fat as a systemic pro-inflammatory signal in CVD risk in NAFLD patients. Further work is required to elucidate the pathophysiological mechanisms explaining the association between free radical-induced oxidative stress and CVD in a NAFLD setting. Clinical evidence could be sought by measuring the plasma levels of FGA, FGB and FGG in NAFLD patients, to investigate potential relationships between plasma fibrinogen levels and severity of CVD determined by conventional atherosclerotic markers, such as carotid artery wall thickness. Indeed, verification of FGA, FGB and FGG as early plasma predictors of CVD development would provide a rationale to explore the possibility of prophylactic fibrinolytic therapy in a NAFLD context, similar to that already used in myocardial infarction [41].

## Additional files

Additional file 1:
**Functional partners of FGA as predicted by STRING analysis.**
Additional file 2:
**Functional partners of FDFT1 as predicted by STRING analysis.**
