# Erratum to: Fibrinogen production is enhanced in an in-vitro model of non-alcoholic fatty liver disease: an isolated risk factor for cardiovascular events?

**DOI:** 10.1186/s12944-016-0290-8

**Published:** 2016-08-01

**Authors:** Emily N. W. Yeung, Philipp Treskes, Sarah F. Martin, Jonathan R. Manning, Donald R. Dunbar, Sophie M. Rogers, Thierry Le Bihan, K. Ann Lockman, Steven D. Morley, Peter C. Hayes, Leonard J. Nelson, John N. Plevris

**Affiliations:** 1 Hepatology Laboratory, Division of Health Sciences, University of Edinburgh, Chancellor’s Building, 49 Little France Crescent, Edinburgh, EH16 4SB UK; 2 Kinetic Parameter Facility, SynthSys, Centre for Synthetic and Systems Biology, University of Edinburgh, C.H. Waddington Building, The Kings Buildings, Edinburgh, EH9 3JD UK; 3 Bioinformatics Team, University/BHF Centre for Cardiovascular Science, University of Edinburgh, Queen’s Medical Research Institute, 47 Little France Crescent, Edinburgh, EH16 4TJ UK

## Erratum

After publication of the original article [1], it came to the authors’ attention that sources of funding for the study presented were inadvertently omitted. The Acknowledgements section should therefore have read as follows:

‘This work was supported by the Biotechnology and Biological Sciences Research Council [grant number BB/L023687/1]. JRM & DRD were supported by the British Heart Foundation Centre of Research Excellence award RE/13/3/30183. The decision to submit the article for publication was not influenced by the funding bodies.’
